# Reproductive plasticity of Hawaiian *Montipora* corals following thermal stress

**DOI:** 10.1038/s41598-021-91030-8

**Published:** 2021-06-09

**Authors:** E. Michael Henley, Mariko Quinn, Jessica Bouwmeester, Jonathan Daly, Nikolas Zuchowicz, Claire Lager, Daniel W. Bailey, Mary Hagedorn

**Affiliations:** 1 Smithsonian Conservation Biology Institute, Front Royal, VA USA; 2 Hawaiʻi Institute of Marine Biology, University of Hawaiʻi at Mānoa, Kāneʻohe, HI USA

**Keywords:** Reproductive biology, Animal physiology, Phenology, Conservation biology, Marine biology, Coral reefs

## Abstract

Ocean warming, fueled by climate change, is the primary cause of coral bleaching events which are predicted to increase in frequency. Bleaching is generally damaging to coral reproduction, can be exacerbated by concomitant stressors like ultraviolet radiation (UVR), and can have lasting impacts to successful reproduction and potential adaptation. We compared morphological and physiological reproductive metrics (e.g., sperm motility, mitochondrial membrane integrity, egg volume, gametes per bundle, and fertilization and settlement success) of two Hawaiian *Montipora* corals after consecutive bleaching events in 2014 and 2015. Between the species, sperm motility and mitochondrial membrane potential had the most disparate results. Percent sperm motility in *M. capitata*, which declined to ~ 40% during bleaching from a normal range of 70–90%, was still less than 50% motile in 2017 and 2018 and had not fully recovered in 2019 (63% motile). By contrast, percent sperm motility in *Montipora spp*. was 86% and 74% in 2018 and 2019, respectively. This reduction in motility was correlated with damage to mitochondria in *M. capitata* but not *Montipora spp*. A major difference between these species is the physiological foundation of their UVR protection, and we hypothesize that UVR protective mechanisms inherent in *Montipora spp*. mitigate this reproductive damage.

## Introduction

Coral reefs are living, dynamic ecosystems experiencing substantial levels of degradation due to human impacts. Globally, increased levels of greenhouse gasses are warming the oceans, making them more acidic, and causing coral to stress, bleach, and have higher susceptibility to newly emergent diseases^1–4^. Locally, reefs are impacted by pollution and sedimentation from poor land-use practices, terrestrial runoff, and destructive fishing practices^5–10^. Additionally, climate change appears to be contributing to increased penetration of ultraviolet radiation (UVR), creating interactive effects with increasing temperatures and high irradiance that further exacerbate environmental stresses^11–15^. Coral bleaching, a stress response involving the loss of a coral colony’s endosymbiotic dinoflagellates, Symbiodiniaceae, can be triggered by any number or combination of stressors such as lowered salinity, increased irradiance and UVR, reduced light from sedimentation and turbidity and more^12,16–18^. Currently, the primary driver of wide-ranging, repeated bleaching events is thermal stress from ocean warming^19^.

Warming can affect corals in several ways. During sustained periods of elevated thermal stress, the loss of a coral colony’s symbionts causes that colony to ‘bleach’—turn pale, pastel, or white in color—and is one of the primary visual impacts associated with increasing ocean temperatures; bleaching is also a significant contributor to large-scale coral mortality^20–24^. If corals survive bleaching, sustained warming periods may impact their reproduction, larval development, recruitment, and survival. For example, elevated temperatures resulted in reduced number and size of oocytes and/or fewer polyps with eggs and testes^25–28^; gametogenesis and spawning may also be repressed the next season or even several seasons after bleaching^25,29^. Additionally, ocean warming can lead to negative impacts in fertilization, larval development, larval survival, pelagic duration, and post-settlement survival^23,30–34^. Increasing temperatures might also shift timing of coral reproduction on a larger temporal scale^28,35^. In contrast, some species appear to be minimally impacted by warming^27,33,34,36,37^.

Multi-year studies on more sensitive species have revealed both immediate and lasting impacts on reproduction due to ocean warming. Following a bleaching event during a long-term study of *Montastraea annularis* (now *Orbicella annularis*), Mendes and Woodley^38^ found that severely bleached colonies did not spawn the next year; gonads were large but reduced in number even two years later in some colonies. Another long-term study of *O. annularis* and *O. franksi* that spanned 12 years and encompassed two bleaching events found that the probability of spawning for both bleached and unbleached corals was significantly reduced, not only during the bleaching event but also 2–3 years after the event, and spawning at the population level had a 4–5 year recovery time^39^.

The usual method for evaluating the health of corals has been by visual assessment—coloration, presence of bleaching/paling, macroalgal competition, and signs of tissue necrosis, skeletal tissue anomalies, lesions, and disease. Nevertheless, environmental fluctuations and perturbations and reduction in coastal water quality can cause sub-lethal stress that cannot be observed by visual surveys alone^40–45^. Global stressors, such as increasing ocean temperatures, may be additive with local stressors which can lead to and/or exacerbate sub-lethal stress that negatively impacts reproduction. If bleaching events continue to occur more frequently and lead to a reduced recovery time, visual observations alone likely miss other responses to these stressors. While visual assessments can be a helpful, rapid, and low-cost method for documenting and reporting a reef’s condition, this method might not accurately capture the state of coral health in that population since it appears that warming events can yield long-lasting, sub-lethal deleterious physiological consequences, especially for reproduction, in both visually bleached and unbleached colonies.

This study was conducted in Kāneʻohe Bay, Hawaiʻi where, historically, mass bleaching incidents were rare. In 2014 and 2015, consecutive sustained warming events, exacerbated by reduced trade winds that increased penetrance of irradiance and UVR exposure, resulted in bleaching of an estimated 45% and 30%, respectively, of coral in the bay^46–48^. The warming trend persisted into 2016 potentially causing further stress, although not visible by eye. Long-term monitoring of reproductive characteristics for two common corals in the bay, *Lobactis scutaria* (formerly *Fungia scutaria*) and *Montipora capitata*, found approximately 50% loss of sperm motility during and after those bleaching events^49^. While recent observations suggest that corals in Kāneʻohe Bay appear to be visually healthy and robust compared to those outside the bay^50^, it was unknown whether their sexual reproduction may have been significantly impacted, at least in part, due to elevated temperatures.

To further understand the mechanisms and response of corals to climate change, comparative studies were conducted to examine their reproductive patterns. We investigated the reproductive traits of the stony coral *Montipora capitata* (Dana 1846), one of the most abundant species in Kāneʻohe Bay with a wide habitat distribution, and the congeneric blue, encrusting corals, *Montipora flabellata* (Studer 1901) and *Montipora dilatata* (Studer 1901), that have a more narrow distribution and are typically restricted to shallow water with high wave energy and irradiance^51,52^. All are broadcasting, simultaneous hermaphrodites that release egg-sperm bundles^53^. With some discrepancy between morphological identification and molecular analysis, it is currently undecided whether *M. flabellata* and *M. dilatata* are separate or the same species^54–57^. Therefore, for this work, all *Montipora* corals that exhibited an encrusting growth form with blue-purple coloration will be referred to as *Montipora spp*. and contrasted against the congener *M. capitata.*

An abundant species, *M. capitata* has a rich and varied publication history spanning decades, and its reproduction is well documented^36,49,58–62^. Conversely, very little was known about the reproduction of the encrusting *Montipora spp*. coral species^53,61,63^. Our preliminary results from 2017 suggested that *Montipora spp.*, with high sperm motility, appeared to be relatively unaffected by the bleaching in 2014 and 2015 whereas *M. capitata* motility had not yet recovered. Toward that end, we made further detailed comparative analyses of the reproductive traits between the well-documented *M. capitata* and the lesser-studied blue *Montipora spp*. in Kaneʻōhe Bay over two consecutive seasons (2018 and 2019) by examining their reproductive morphological characteristics (e.g. sperm concentration, number of eggs per bundle, and egg volume), their cellular physiological characteristics with Computer Assisted Sperm Analysis (percent sperm motility), and their subcellular physiological characteristics with flow cytometry (mitochondrial membrane potential). Finally, we examined their ability to fertilize and settle.

## Results

**Figure 1:**
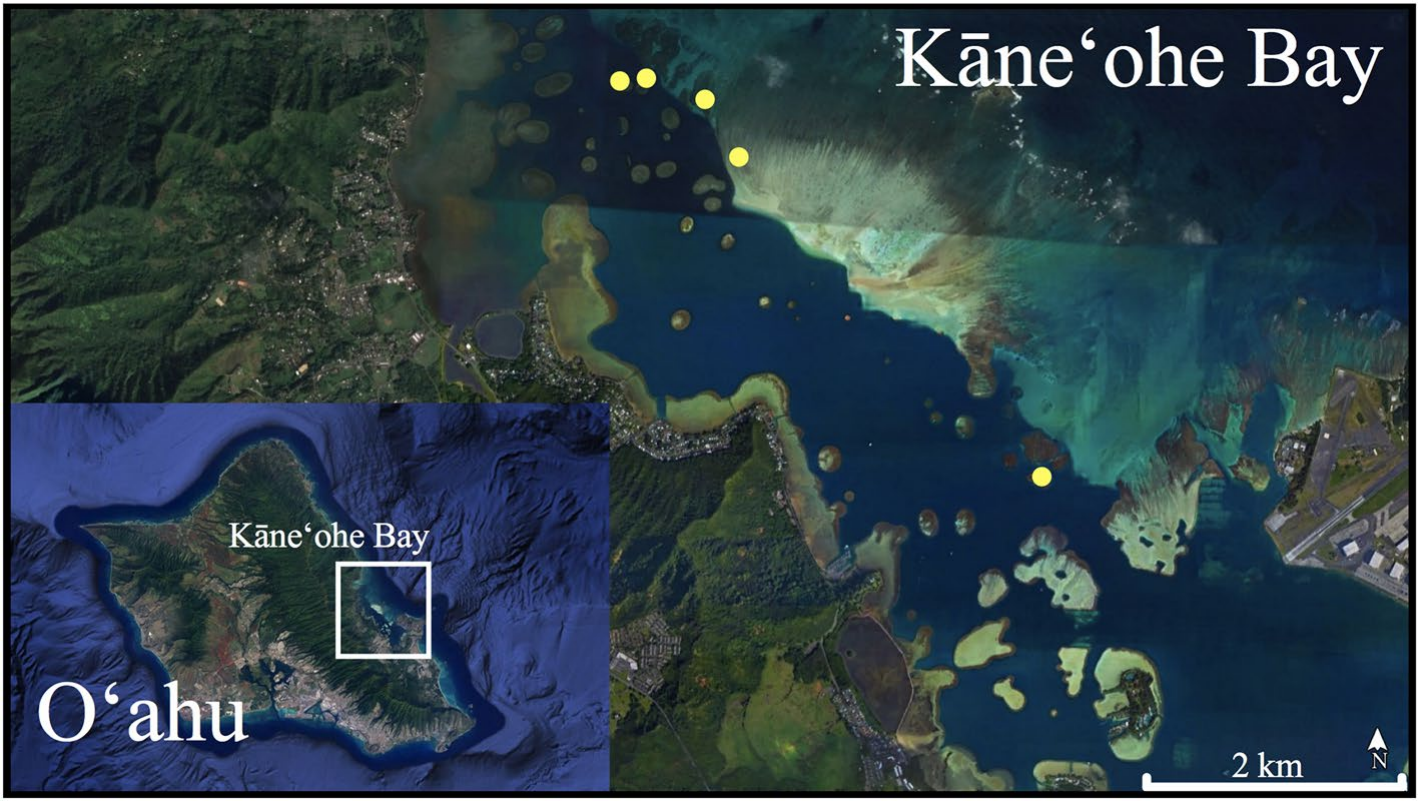
Collection sites for *M. capitata* and *Montipora spp*. Colonies were collected across the bay between depths of 1–4 m (*Google Earth Pro* v. 7.3.3.7786, www.earth.google.com).

The following comparative experiments explored the differences in some of the reproductive characteristics for two Hawaiian scleractinian corals—*M. capitata*, which has been substantially studied, and *Montipora spp.* which has been minimally studied—years after consecutive ocean warming and bleaching events. Reproduction data were collected during the summer spawning season of June to August/September of 2018 and 2019 from colonies of *Montipora capitata* and *Montipora spp.* found throughout Kāneʻohe Bay, Hawaiʻi (Fig. 1).

Overall, *Montipora spp*. appeared to perform better and more consistently than *M. capitata* in most of the reproductive metrics assessed: sperm motility and mitochondrial membrane potential, fertilization, and larval settlement (Table 1). We found that, comparatively, *M. capitata* improved in all assessed reproductive metrics from 2018 to 2019, except fertilization where it declined; settlement in 2019 was the lone metric where *M. capitata* performed marginally better than *Montipora spp*. Some preliminary spawning data of comparative sperm motility estimates were previously recorded during the summer of 2017 (Table 1). Whereas *M. flabellata* and *M. dilatata* have historically been classified as two species, there has not yet been a formal revision of Hawaiian Montipora taxonomy. Therefore, the *Montipora spp*. colonies for these experiments were originally visually identified and labeled as *M. flabellata* and *M. dilatata*, and those distinctions were maintained throughout the duration of the study. We initially compared fertilization success between combinations of crosses of *M. flabellata* and *M. dilatata* and found no difference in their ability to fertilize (Supplementary Fig. S1). Based upon this and previous genetic analysis, the two species were combined to compare to *M. capitata*.

**Table 1:** Summary of reproductive data.

Metric	*M. capitata*	*Montipora spp*.
Eggs per bundle	12.3 ± 0.2875 N = 8; n = 156	8.7 ± 0.213 N = 12; n = 156
Sperm concentration per bundle (mL)	1.5 × 10^6^ cells/mL ± 1.2 × 10^5^ N = 8; n = 142	7.5 × 10^5^ cells/mL ± 5.1 × 10^4^ N = 12; n = 142
Egg volume	0.04076 mm^3^ ± 0.00033 N = 16; n = 564	0.03903 mm^3^ ± 0.00055 N = 14; n = 564
Sperm motility	2017: 42% ± 8.1% N = 11 Pooled; n = 11 2018-Initial: 47% ± 2.1% N = 9; n = 55 2018-1Hr: 38% ± 5.1% N = 8; n = 31 2019-Initial: 63% ± 3.01% N = 16; n = 23 2019-1Hr: 64% ± 3.8% N = 16; n = 23	2017: 58% ± 7.1% N = 9; n = 16 2018-Initial: 84% ± 0.96% N = 16; n = 137 2018-1Hr: 82% ± 1.16% N = 16; n = 136 2019-Initial: 74% ± 2.1% N = 14; n = 61 2019-1Hr: 71% ± 2.07% N = 14; n = 61
Sperm with high mitochondrial membrane potential	2018: 36% ± 2.46% N = 9; n = 55 2019: 65% ± 1.86% N = 16; n = 23	2018: 78% ± 1.37% N = 16; n = 50 2019: 81% ± 1.2% N = 14; n = 61
Correlation of sperm motility and high membrane potential	2018: R^2^ = 0.1025; *p* = 0.0172 N = 9; n = 55 2019: R^2^ = 0.3715; *p* = 0.002 N = 16; n = 23	2018: R^2^ = 0.2160; *p* < 0.0001 N = 16; n = 137 2019: R^2^ = 0.3655; *p* < 0.0001 N = 14; n = 61
Fertilization	2018: 51% ± 5.71% N = 12; n = 14 2019: 20% ± 4.1% N = 19; n = 19	2018: 56% ± 5.09% N = 13; n = 18 2019: 60% ± 3.56% N = 26; n = 30
Settlement	2018: 7% ± 1.45% N = 7; n = 36 2019: 23% ± 4.92% N = 19; n = 18	2018: 16% ± 2.31% N = 19; n = 56 2019: 11% ± 1.81% N = 24; n = 55

Reproductive metrics assessed for *Montipora capitata* and *Montipora spp*. in 2018 and 2019 with mean and SEM. Number of unique genotypes (N), unique crosses (N), and sample replicates (n) used per analysis and year are given. Unique crosses are genotype combinations used for fertilization and settlement only. The coefficient of determination (R^2^) is given for the proportion of variance in percent sperm motility explained by the variation in percent of sperm with high mitochondrial membrane integrity.

### Eggs and sperm concentration per bundle

**Figure 2:**
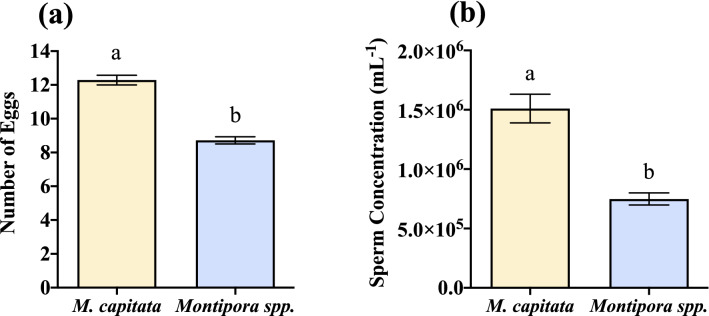
Eggs and sperm per bundle. (**a**) The mean number eggs per bundle is greater in *Montipora capitata* than *Montipora spp*. (n = 156 M*. capitata*, n = 156 *Montipora spp*., *p* < 0.0001, Welch’s t = 9.979). (**b**) Mean sperm concentration per mL per bundle is also greater in *Montipora capitata* than *Montipora spp*. (n = 142 M*. capitata*, n = 142 *Montipora spp*., *p* < 0.0001, t = 5.45). Error bars are SEM, and different letters above error bars indicate significant difference. (GraphPad Prism 9 v. 9.0.1; San Diego, CA, USA, www.graphpad.com).

Fecundity can be used as a comparative reproductive health metric, and one commonly used method is to determine the number of eggs and concentration of sperm in each bundle that is released. With larger bundles, *M. capitata* has nearly 30% more eggs per bundle than *Montipora spp.* (Table 1, Fig. 2a; *p* < 0.0001, Welch’s t = 9.979), and *M. capitata* has 50% more sperm per bundle than *Montipora spp.* (Table 1, Fig. 2b; *p* < 0.0001, t = 5.45). However, the polyp density of *Montipora spp*. (~ 75 polyps/cm^2^) is about three times greater than that of *M. capitata* (~ 25 polyps/cm^2^), while the diameter of the corallite for *Montipora spp*. and *M. capitata* is about 1 mm and 2 mm, respectively. Since *M. capitata* has fewer but larger polyps per cm^2^, it’s perhaps not surprising that it also produces larger egg-sperm bundles, with more eggs and sperm per bundle than *Montipora spp.*

There were other gross morphological differences, as well, related to the integrity or cohesion of the bundles. After the bundles were collected and placed in 15 mL tubes, the break-up time for bundles was also noted. In general, *M. capitata* seemed to have robust bundles that typically took about 45–60 min to break apart after initial spawning. By contrast, *Montipora spp*. bundles would frequently take less than 30 min to break apart to release the eggs and sperm.

### Egg volume

**Figure 3:**
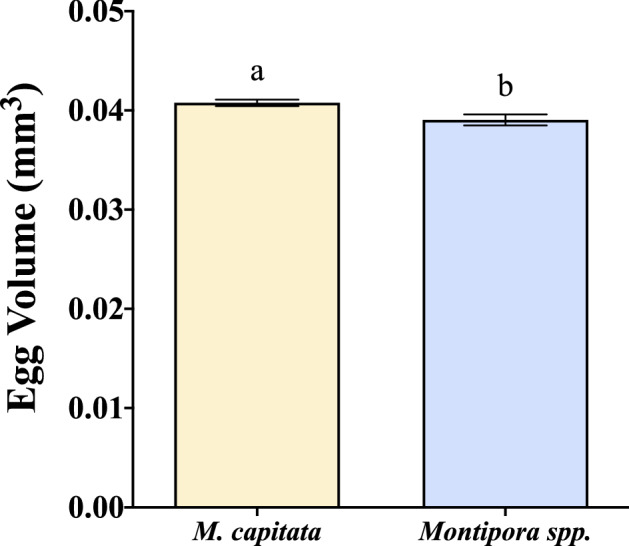
Egg volume compared between *M. capitata* and *Montipora spp*. The difference in egg volume (mm^3^) between *Montipora capitata* and *Montipora spp*. on night of spawning after bundles had broken apart and hydrated for 30 min. The mean volumes were similar; however, there was greater variation in the volume of *Montipora spp*. eggs, possibly leading to the significant difference (n = 564 M*. capitata*, n = 564 *Montipora spp*., *p* = 0.0002, Welch’s t = 3.791). Error bars are SEM, and different letters above error bars indicate significant difference. (GraphPad Prism 9 v. 9.0.1; San Diego, CA, USA, www.graphpad.com).

Egg size is another comparative reproductive health metric used as a proxy for energy investment (via lipids) into reproduction by the parent colony. Despite the smaller bundle size of *Montipora spp*., their individual egg volume was not appreciably different than *M. capitata*. The mean egg volume of *M. capitata* is greater than the mean egg volume of *Montipora spp*., but only with an approximately 4% difference (Table 1, Fig. 3; *p* = 0.0002, Welch’s t = 3.76). This difference may be supported by greater variability in egg volume observed in *Montipora spp*. and its extended gametogenic and spawning cycle.

### Sperm motility

**Figure 4:**
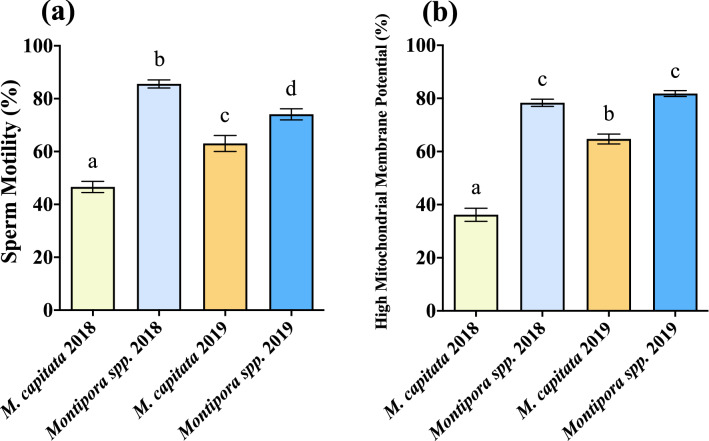
Percent sperm motility and percent of sperm with high mitochondrial integrity. (**a**) The difference in mean percent total sperm motility (2018: n = 55 M*. capitata*, n = 50 *Montipora spp*.; 2019: n = 23 M*. capitata*, n = 61 *Montipora spp*.; see Table S2 in Supplementary Information for two-way ANOVA results). While sperm motility declined in *Montipora spp*. and improved in *M. capitata* from 2018 to 2019, total motility was still greater in *Montipora spp.* than *M. capitata* both years. (**b**) The percent of sperm with high mitochondrial membrane potential (2018: n = 55 M*. capitata*, n = 50 *Montipora spp*.; 2019: n = 23 M*. capitata*, n = 61 *Montipora spp*.; see Table S3 in Supplementary Information for two-way ANOVA results). The percent of sperm with high mitochondrial membrane integrity maintained a similar level in *Montipora spp*., and while it significantly improved in *M. capitata* from 2018 to 2019, *Montipora spp*. was significantly greater both years. Error bars are SEM, and shared letters above error bars indicate no significant difference as determined by post hoc test of two-way ANOVA. (GraphPad Prism 9 v. 9.0.1; San Diego, CA, USA, www.graphpad.com).

One of the greatest differences between the species was observed in their sperm motility, with *Montipora spp.* having consistently more motile sperm, and in order to quantify sperm motility, a Computer Assisted Sperm Analysis (CASA) software system was used in conjunction with computer-aided video microscopy. Overall, the mean percent sperm motility differed for the two species in each year (Table 1, Fig. 4a). *Montipora spp.* total motility was nearly double that of *M. capitata* in 2018 (84% and 47%, respectively). *Montipora capitata* sperm had much higher total motility in 2019 (63%) but still lower than that of *Montipora spp.* (74%). The mean percent sperm motility for *Montipora spp.* was greater in 2018 than in 2019, while the reverse was true for *M. capitata* (Table 1, Fig. 4a). A two-way ANOVA determined that there was a significant interaction between species and year (F = 36.64, *p* < 0.0001). Species (F = 189.34, *p* < 0.0001) and year (F = 14.52, *p* = 0.0002) are both significant factors, but species had a stronger effect on sperm motility (Supplementary Table S2).

**Figure 5:**
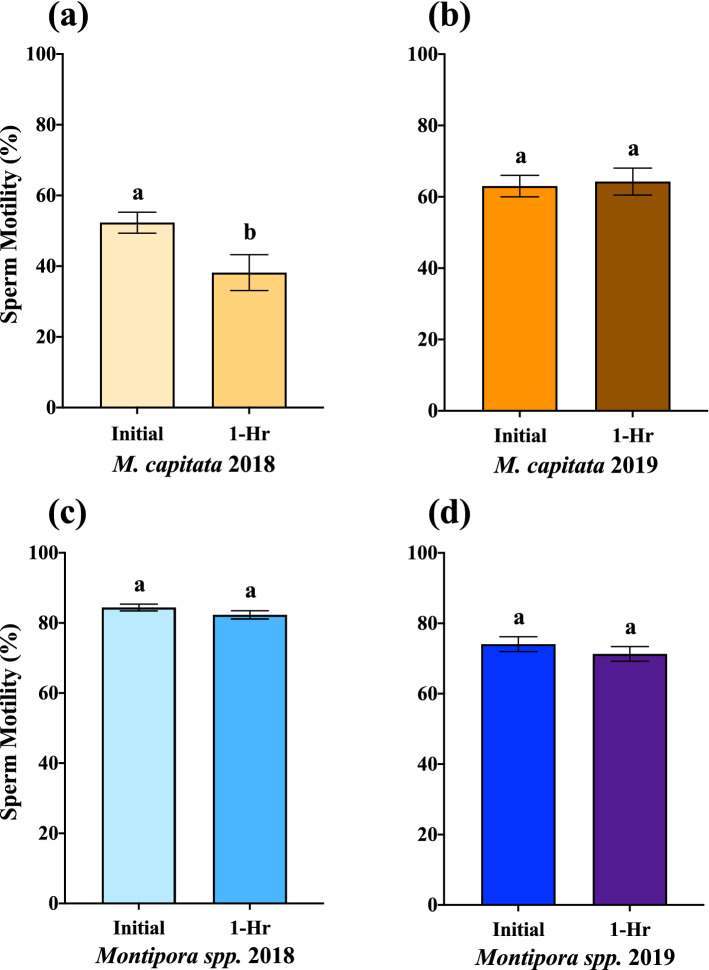
Initial percent sperm motility and 1-Hour reassessment. The difference in initial mean percent sperm motility shortly after bundles had broken apart and one hour after initial assessment for both species each year. (**a**) *M. capitata* 2018 (n = 31 Initial, n = 31 1-Hr, *p* = 0.0041, Welch’s t = 3.03). (**b**) *M. capitata* 2019 (n = 23 Initial, n = 23 1-Hr, *p* = 0.7089, Welch’s t = 0.3759). (**c**) *Montipora spp*. 2018 (n = 136 Initial, n = 136 1-Hr, *p* = 0.2296, Welch’s t = 1.204). (**d**) *Montipora spp*. 2019 (n = 61 Initial, n = 61 1-Hr, *p* = 0.2855, Welch’s t = 1.073). Percent sperm motility maintained similar levels after an hour of swimming in *Montipora spp*. both years and *M. capitata* in 2019, whereas motility in *M. capitata* 2018 declined over an hour. Error bars are SEM, and different letters above error bars indicate significant difference. (GraphPad Prism 9 v. 9.0.1; San Diego, CA, USA, www.graphpad.com).

Additionally, there were differences in the longevity of sperm motility for *M. capitata* but not for *Montipora spp*. In 2018, *M. capitata* motility declined approximately 10% over one hour (Table 1, Fig. 5a, p = 0.0041, Welch’s t = 3.03) but maintained its motility in 2019 (Table 1, Fig. 5b, p = 0.7089, Welch’s t = 0.3759). *Montipora spp*. sustained its motility over an hour with similar levels in both 2018 (Table 1, Fig. 5c, p = 0.2296, Welch’s t = 1.204) and 2019 (Table 1, Fig. 5d, p = 0.2855, Welch’s t = 1.073).

### Mitochondrial membrane potential

The key energy source for sperm motility resides in the mitochondria. Mitochondrial respiration, fueled by a pH gradient, yields a mitochondrial membrane potential that details the energetic state of the cells; low potential indicates less energy and high potential indicates higher energy and is correlated with sperm motility^64–66^. Therefore, flow cytometry analysis of the percentage of sperm with high mitochondrial membrane potential was also used as an indicator of healthy sperm cells. Mirroring motility, the patterns of mitochondrial integrity were similar with *Montipora spp*. (2018: 78%; 2019: 81%) having a greater percentage of sperm with high membrane potential than *M. capitata* (2018: 36%; 2019: 65%) for both years (Table 1, Fig. 4b). A two-way ANOVA determined that species (F = 262.73, *p* < 0.0001), year (F = 61.46, *p* < 0.0001), and interaction between species and year (F = 25.8, *p* < 0.0001) were all significant factors (Supplementary Table S3).

In both 2018 and 2019, *Montipora spp*. outperformed *M. capitata* in terms of both total sperm motility and percent of sperm with high mitochondrial membrane potential. Both of these metrics in *M. capitata* were much lower in 2018 but rebounded in 2019, though still not performing as well as *Montipora spp.* (Table 1, Fig. 4). The results of the two-way ANOVA for both motility and mitochondrial integrity indicate that the interaction of species and year was a significant factor, and species had a significant main effect for both. While year alone did not have a significant effect on sperm motility, it had a significant effect on mitochondrial integrity; however, given the relatively large F-ratios for species in both analyses, it is likely that species was the primary driver for differences in the analysis (Supplementary Tables S2 and S3).

### Correlation of sperm motility and mitochondrial membrane potential

**Figure 6:**
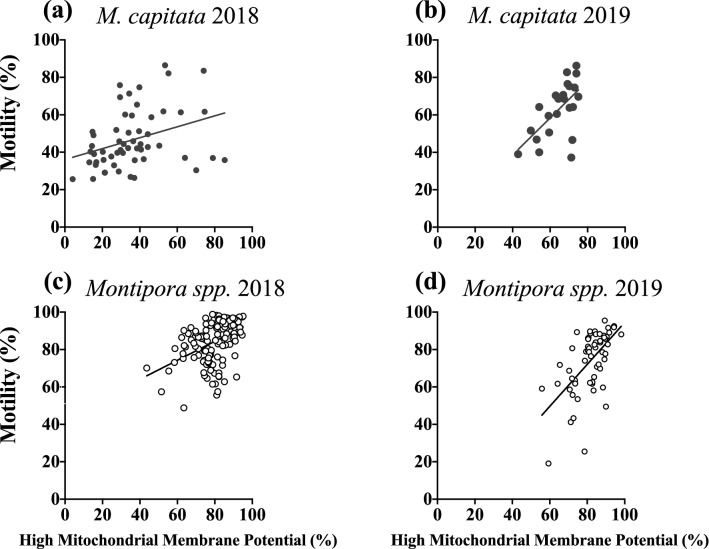
Correlation of percent sperm motility with percent of sperm with high mitochondrial integrity. (**a**) In 2018 M*. capitata*, approximately 10% of the variation is shared between motility and membrane potential (n = 55, R^2^ = 0.1025, *p* = 0.0172). (**b**) In 2019 M*. capitata*, that increased to 37% of the variance shared (**b** n = 23, R^2^ = 0.3715, *p* = 0.002)*.* (**c**) In 2018 *Montipora spp.*, about 22% of the variance is shared between motility and membrane integrity (n = 137, R^2^ = 0.2160, *p* < 0.0001). (**d**) In 2019 *Montipora spp.*, that increased to 37% of the variance shared between the two (n = 61, R^2^ = 0.3655, *p* < 0.0001). (GraphPad Prism 9 v. 9.0.1; San Diego, CA, USA, www.graphpad.com).

There was a significant correlation between percent sperm motility and percent of sperm with high membrane potential for both species in each year, but the strength of that correlation varied for each season. For *M. capitata* in 2018, about 10% of the variance is shared between motility and membrane integrity (Table 1, Fig. 6a; R^2^ = 0.1025, *p* = 0.0172). In 2019 there was a stronger correlation found in *M. capitata* with 37% of the variance shared between motility and membrane integrity (Table 1, Fig. 6b; R^2^ = 0.3715, *p* = 0.002). A similar pattern was seen among the years in *Montipora spp.* In 2018 about 22% of the variance was shared between motility and membrane integrity (Table 1, Fig. 6c; R^2^ = 0.2160, *p* < 0.0001) and in 2019 37% of the variance shared between motility and membrane integrity (Table 1, Fig. 6d; R^2^ = 0.3655, *p* < 0.0001). The quality of *Montipora spp.* sperm (motility and membrane integrity) outperformed that of *M. capitata* both years. Additionally, correlation of sperm motility with membrane potential for each species and year suggests that a significant positive relationship between the two exists, though the correlation appears to be relatively weak, indicating that other factors could have been influencing sperm motility.

### Fertilization

**Figure 7:**
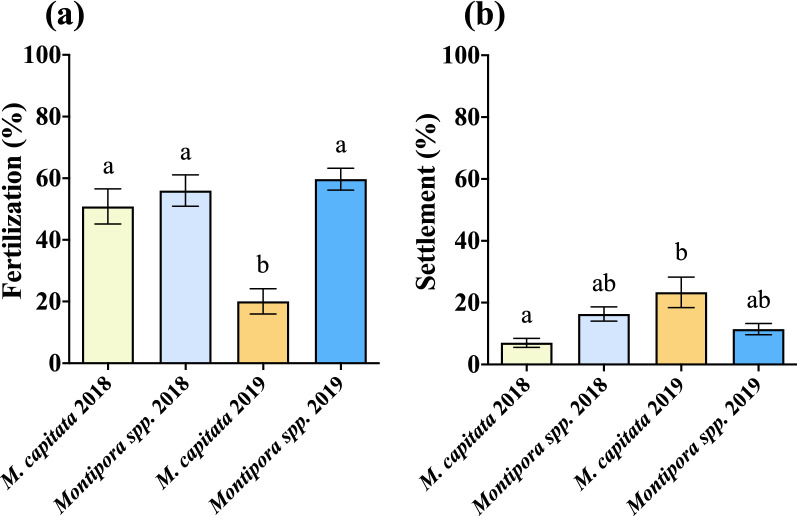
Percent fertilization and percent settlement. (**a**) The difference in mean percent fertilization (2018: n = 14 M*. capitata*, n = 18 *Montipora spp*.; 2019: n = 19 M*. capitata*, n = 30 *Montipora spp*.; see Table S4 in Supplementary Information for two-way ANOVA results). Fertilization success significantly declined in *M. capitata* from 2018 to 2019 while remaining consistent in *Montipora spp*. There was no difference in fertilization success between the two in 2018, but there was a noticeable significant difference in 2019. (**b**) The difference in mean percent settlement between *M. capitata* and *Montipora spp*. (2018: n = 36 M*. capitata*, n = 56 *Montipora spp*.; 2019: n = 18 M*. capitata*, n = 55 *Montipora spp*.; see Table S5 in Supplementary Information for two-way ANOVA results). For *M. capitata*, there was an increase in settlement percent from 2018 to 2019, but there was no difference between 2018 and 2019 for *Montipora spp*. Settlement was higher for *Montipora spp.* than *M. capitata* in 2018, and this was reversed in 2019. Error bars are SEM, and shared letters above error bars indicate no significant difference as determined by post hoc test of two-way ANOVA. (GraphPad Prism 9 v. 9.0.1; San Diego, CA, USA, www.graphpad.com).

Successful fertilization was examined using bundle–bundle crosses in scintillation vials. *Montipora spp*. maintained relatively high fertilization success in both years while *M. capitata* declined from 2018 to 2019 (Table 1, Fig. 7a). Overall, percent fertilization success was similar between *M. capitata* (51%) and *Montipora spp*. (56%) in 2018 and again for *Montipora spp*. in 2019 (60%). Fertilization for *M. capitata* in 2019 (20%) declined substantially from the previous year. No self-fertilization was observed. The results of the two-way ANOVA for fertilization determined that there was an interaction between species and year (F = 12.63, *p* = 0.0006) which was similar to year alone which also had a significant effect (F = 4.62, *p* = 0.0001). Species did not have a significant main effect (F = 0.584, *p* = 0.4469) on fertilization (Supplementary Table S4).

Fertilization success was only marginally higher in *Montipora spp*. in 2018 and was similar in 2019 when *M. capitata* considerably declined. Under normal climate conditions and with closed in vitro systems, generally fertilization success can be very high regardless of motility of sperm if sufficient concentrations are applied. For these experiments, sperm concentration was maintained and not altered by orders of magnitude. It may be, then, that some aspect of egg competency in *M. capitata* was responsible for the variable fertilization success, but that was not examined here.

### Settlement

Following development, recruitment for marine larvae is a critical life stage. For *M. capitata*, there was an increase in percent settlement success from 2018 (7%) to 2019 (23%) while *Montipora spp*. slightly declined (16% and 11%, respectively). Settlement was higher for *Montipora spp.* than *M. capitata* in 2018, while the reverse was true in 2019. (Table 1, Fig. 7b). A two-way ANOVA for settlement determined that both species (F = 7.947, *p* = 0.0066) and year (F = 15.79, *p* = 0.0002) alone had significant effects, and there was also a significant interaction between species and year (F = 17.14, *p* = 0.0001) on settlement (Supplementary Table S5).

The settlement trials were performed in six well plates with a small chip of crustose coralline algae (CCA) as a settlement cue. Because *M. capitata* is found over a wider range of habitats than *Montipora spp*. (which seems to be restricted to areas of higher wave energy), settlement cues such as water movement, light intensity, and other physical and chemical parameters are not replicated in a stagnant six well plate and may help explain the low settlement of *Montipora spp*. Considering all the downstream effects that impact larval coral development and the variable habitat where it is found, it may be that *M. capitata* is less restrictive in its preference for settlement than *Montipora spp*., although a different experimental design would be needed to assess that than was utilized here.

## Discussion

**Figure 8:**
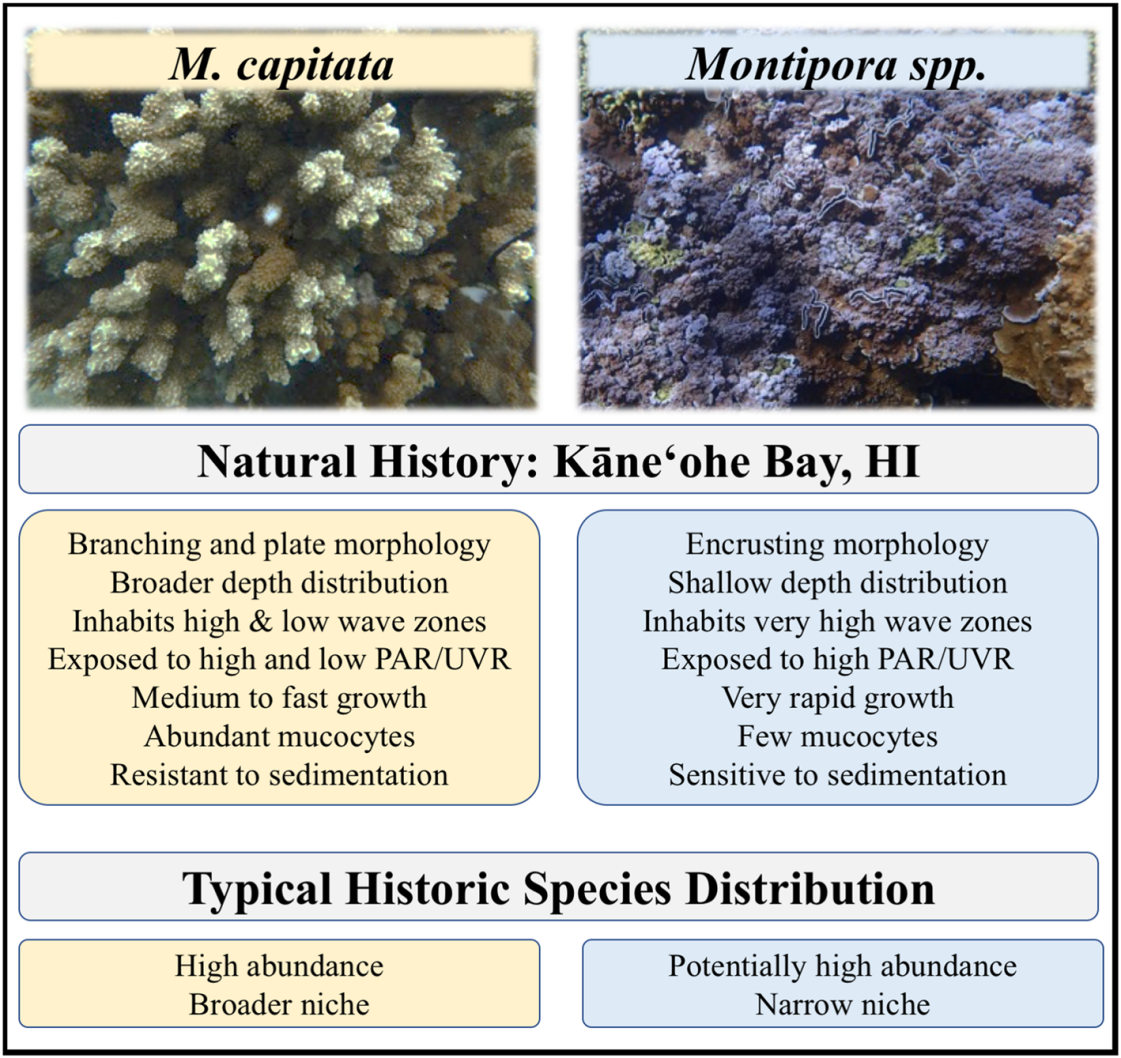
Overview of natural history, traits, and species distribution of *M. capitata* and *Montipora spp*. *Montipora capitata* inhabits a broader range of reef habitat than *Montipora spp*. which is limited to shallow reefs with high wave and light energy.

*Montipora capitata* is one of Hawaiʻi’s main reef-building corals and is vital to reef habitat and growth^51^. Both *Montipora flabellata* and *Montipora dilatata–Montipora spp*.—are Hawaiian endemics that inhabit a much narrower ecological range than *M. capitata*^51,52^. See Fig. 8 for details. While we do not have bleaching histories for the colonies used here, we do have long-term sperm motility data for the *M. capitata* population in Kāneʻohe Bay that encompasses the years prior to bleaching^49^. With its relative scarcity in the bay, *Montipora spp*. is rarely used for reproductive studies, individual colony history is not typically tracked, and no similar long-term reproductive data set exists for *Montipora spp*. Therefore, it is possible that the colonies used here have differences in bleaching and/or recovery from the consecutive bleaching events of 2014 and 2015.

Following bleaching, coral species differ widely—ranging from months to years—in their ability to recover from thermal stress and invest in sexual reproduction^27,33,39,67^. By increasing heterotrophy, *M. capitata* has previously been demonstrated to quickly recover lipid reserves after bleaching, likely allowing it to maintain fecundity, at least in terms of egg production^36,62^. Given the results of our experiments, certain aspects of *M. capitata* reproduction may be more sensitive to environmental perturbations and might take longer to recover than previously thought and, additionally, longer than *Montipora spp*. For example, it appears that *Montipora spp*. maintained higher quality sperm production more consistently and with less variation than *M. capitata*. It might be, then, that sperm production in *M. capitata* is more heavily influenced by sustained temperature extremes, UVR exposure, and/or other stressors than *Montipora spp*.

Many reproductive studies are done by means of histological analysis which can provide a snapshot of reproductive condition at the time of sampling but cannot clearly delineate the dynamics of reproductive physiology. Because oocytes are orders of magnitude larger than sperm and generally more energetically expensive^68,69^, studies following reproductive investment in corals after stress events have primarily focused on maternal colony effects and impacts to oocytes^25–28,36,37,70^. Analysis of sperm—if even examined—is typically limited to presence/absence of mature testes, sperm per bundle, and/or histological measurements^26,28,37^. Using histological processes, Johnston, et al.^71^ suggested that thermal stress in 2014 and 2015 in Kāneʻohe Bay had a greater and longer-term impact on oocyte development than on the testes in *Pocillopora meandrina*, but without active physiology measurements at spawning, it is difficult to fully assess the state of recovery.

One of the most susceptible coral reproductive metrics impacted by ocean warming is sperm sensitivity, exhibited by reduced motility^49,72^. Prior to 2014, *M. capitata* was commonly observed to have sperm motility greater than 70% to 90%^49^. When preliminary data were collected in 2017—two years after the sequential bleaching events of 2014 and 2015—sperm motility in *Montipora spp*. and *M. capitata* was 58%, and 42%, respectively (Table 1). In 2018, three years later, *M. capitata* sperm motility was still comparable to levels observed at the height of bleaching in 2015^49^, suggesting that its motility had not markedly recovered until 2019, four years after the consecutive bleaching events, yet still had not returned to levels observed prior to 2014 (Table 1, Fig. 4a).

Unfortunately, no similar long-term data for *Montipora spp.* reproduction exist for comparison, as this species has not been the target for this type of reproductive research, but after a relatively high preliminary motility assessment in 2017, *Montipora spp*. sperm motility outperformed *M. capitata* the following two years (Table 1, Figs. 4a, 5). Additionally, mitochondrial potential mirrored motility assessments in both 2018 and 2019, with *M. capitata* improving and *Montipora spp*. maintaining an already high level by comparison (Table 1, Fig. 4b). This suggests that if motility and mitochondrial membrane potential were impacted from bleaching in 2014 and 2015, *Montipora spp*. recovered more quickly than *M. capitata*, possibly in half the time, or perhaps it was never impacted at all by those events.

Understanding sperm characteristics and physiology is important, especially in light of climate change’s impact on and the response to reproduction. But in a larger context, are marine populations sperm-limited? This is largely going to depend on the environment, life history traits, and current population status of the taxon in question. As reviewed by Levitan and Petersen^73^, percent fertilization in marine organisms increases due to proximity, population density and size, and number of gametes released and declines with gamete dilution via current velocity. In his review, Yund^74^ indicates that mobile and pair-bonding organisms, like some fish species, have very high fertilization success and are likely not sperm limited while benthic, sessile, and sedentary broadcasting invertebrates have some of the lowest rates of successful fertilization; however, there are documented cases of broadcasting invertebrates with high fertilization rates. Additionally, there are adaptations to enhance fertilization—synchronous spawning, sperm storage, larger egg size, sperm chemotaxis, and increased motile longevity—and mechanisms to prevent polyspermy^74^. Field measurements combined with in vitro fertilization assessments in corals, urchins, and sea stars have suggested that sperm limitation is a factor in some populations and could be due to limited sperm energy reserves, gamete dilution, proximity of conspecifics, and changes in sperm:egg ratio, but there are also life-history traits to reduce these effects^75–80^.

Considering coral reproduction and increasing ocean temperatures, Omori, et al.^72^ reported a decline in sperm motility and fertilization success in *Acropora* corals following the 1998 worldwide bleaching event; sperm concentrations one and two orders of magnitude greater than prior to bleaching were required to achieve 80% and 95% fertilization, respectively, suggesting that decline in sperm performance rather than competency of eggs was the more significant change. By contrast, Armoza-Zvuloni, et al.^37^ suggested that acclimatization and/or increased heterotrophy in the coral *Oculina patagonica* might explain why there was no difference in developed oocytes and testes between those that had repeatedly bleached and those that had not. However, development of gametes was only investigated histologically; no other metrics of reproduction after spawning were assessed in that study.

Even if populations across taxa were not historically sperm limited, anthropogenic disturbances through overharvesting, pollution, disease, climate change, etc. could potentially reduce populations to levels lower than their evolutionary history, making sperm limitation more likely^74,81^. A reduction in sperm motility accompanying a population decline could, conceivably, further limit successful fertilization and complicate recovery. If certain aspects of *Montipora spp*. reproduction are more robust than *M. capitata*, what might be contributing to that? The combination of high sea surface temperatures and solar irradiance likely exacerbate coral bleaching and/or sublethal stress^15,82,83^. Increased temperatures have a long history of detrimental effects on many aspects of reproduction, but UVR has also been implicated in negative impacts as well. For example, damage to coral gonads^84^, fish sperm chromatin^85^, and urchin sperm mitochondrial membrane damage, DNA and chromatin damage, and morphological abnormalities^86^ all correlate with high UVR.

Shallow water corals inhabit areas of the reef with supersaturating irradiance and high exposure to UVR^87^. Corals in these waters are known to produce UVR photoprotective pigments in their tissues, chromoproteins and fluorescent proteins, and/or concentrate symbiont-produced UVR photoprotective mycosporine-like amino acids (MAAs) in their surface mucus^88–91^. UV-protective chromoproteins in *Montipora spp*. are found colony-wide in a thick band in the upper layer of epidermis, superficial to the nuclei and concentrated at the surface^48^. These chromoproteins are not found in other Hawaiian coral species such as *Porites compressa*, *P. evermanni*, *Pocillopora meandrina*, and *M. capitata*; interestingly, they are found in the polyps but not in the remaining colony tissue of *M. patula*^48^. Chromoproteins and fluorescent proteins are not very energetically costly and would need to be in abundance if serving as photoprotection^89^. The correlation of having higher concentrations of UVR-protective chromoproteins produced by the coral colony has, however, been linked with higher susceptibility to bleaching and mortality^92^, and *Montipora spp.* appears to be extremely sensitive to thermal stress, as observed during the bleaching events in 2014^48,93^. Additionally, Richards Donà^48^ found *Montipora spp*. to be inefficient at removing sediments and histologically is significantly lacking in mucocytes compared to other *Montipora* corals, which could explain why it is restricted to areas of higher water flow. *Montipora spp*. symbionts were also enrobed with UVR-absorbing melanin which was not present in its congener, *M. patula*^48^.

**Figure 9:**
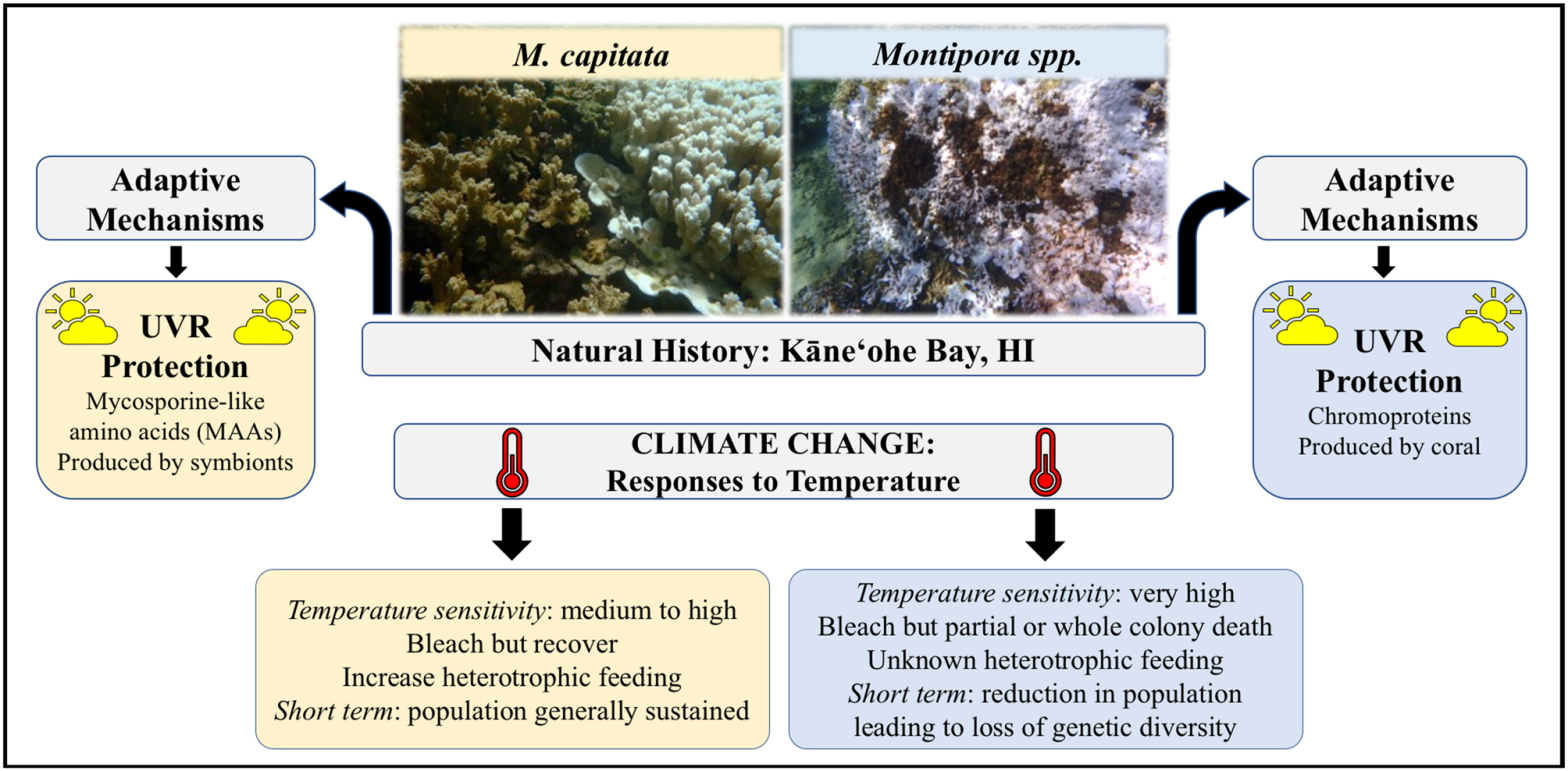
Physiological mechanism of UVR mitigation and response to thermal stress, *M. capitata* and *Montipora spp*. UVR protection for *M. capitata* is derived from MAAs that are produced by its symbionts whereas *Montipora spp*. produces its own chromoproteins that serve as UVR protectant. *Montipora spp*. is also more sensitive to elevated temperatures while *M. capitata* appears to be more robust, possibly by increasing heterotrophic feeding. This strategy seems unlikely for *Montipora spp*.

Taken together, the lack of mucocytes producing very little mucous, likely relying less on the translocation of on MAAs for photoprotection, and instead incorporating endosymbionts enrobed with UVR-absorbing melanin and a thick band of superficial chromoproteins in the epithelium suggests a different strategy to mediate the supersaturated light conditions on shallow reefs^48^. Perhaps this alternative photoprotective strategy also is better at shielding gametes or their associated stem cells during and/or prior to gametogenesis. See Fig. 9 for details.

**Figure 10:**
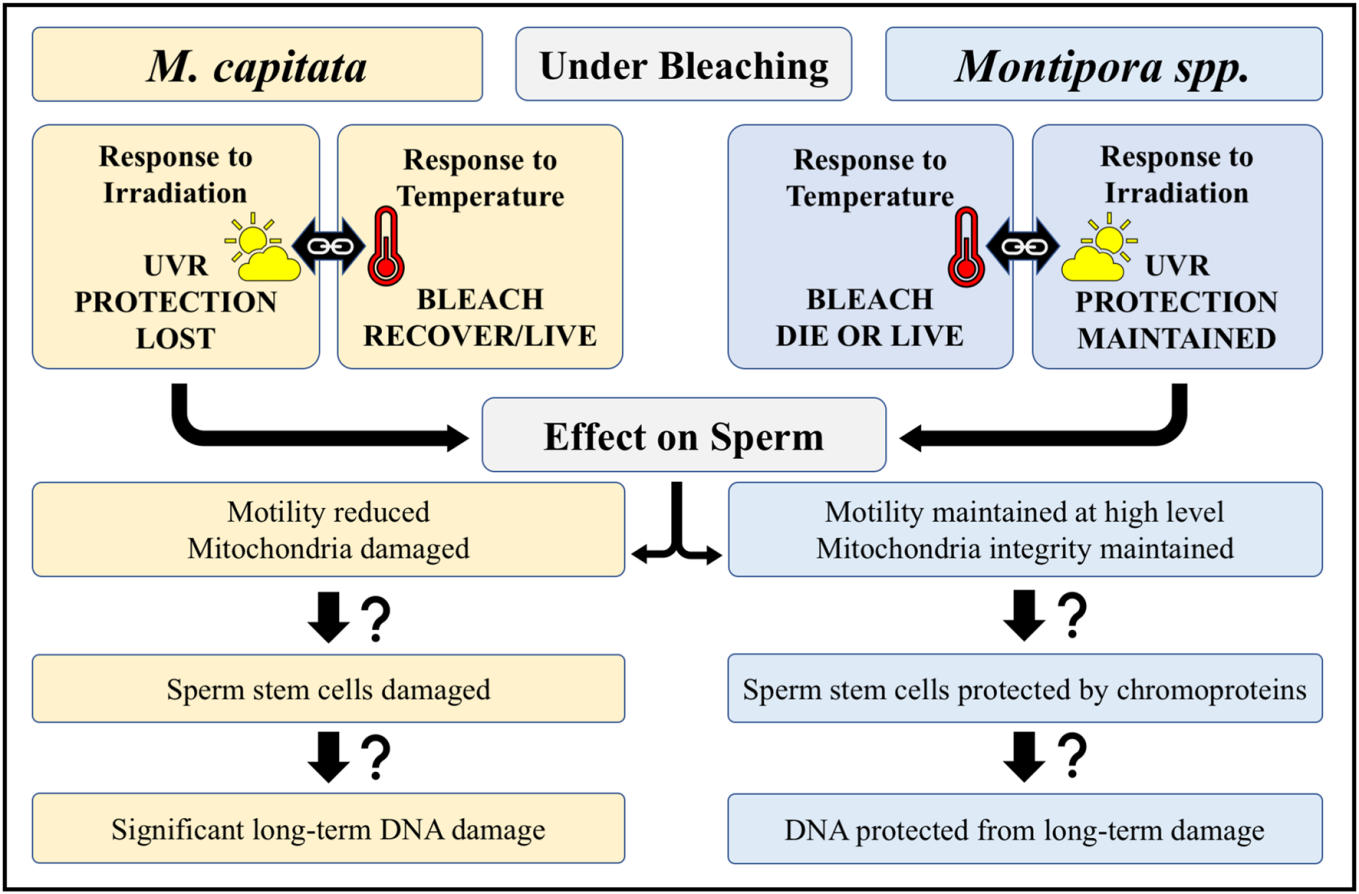
Hypothesized mechanisms of protection (*Montipora spp*.) or damage (*M. capitata*) of sperm from UVR while bleached. While *Montipora spp.* is more likely to experience mortality, if a portion of the colony lives, its reproduction might be protected. Conversely, if the *M. capitata* colony is more likely to recover, its reproduction might sustain longer-term damage due to loss of UVR protection while bleached.

Given that it took *M. capitata* in Kāneʻohe Bay nearly four years to recover sperm motility levels comparable to those observed prior to bleaching, we speculate that UVR might be causing long-term damage to the reproduction of shallow water corals. Our hypothesis as to why reproductive characteristics in *Montipora spp*. were superior to *M. capitata* is that, while bleached, the coral-produced UVR protective chromoproteins observed in *Montipora spp*. might be guarding its stem and/or gametogenic cells from damage while that UVR protection, MAAs produced by its symbionts, is lost in *M. capitata*. See Fig. 10 for details.

Perhaps, then, a trade-off for *Montipora spp.* to bleaching sensitivity and frequent partial colony mortality is highly competitive rapid growth and increased vegetative (asexual) reproduction during periods with normal, lower temperatures and investment in UVR protection of gametes and/or stem cells. While bleached, *Montipora spp*. would presumably retain that UVR protection, thus protecting against any long-term DNA damage. By contrast, *M. capitata* appears to be more robust to bleaching events such that colony death might be less prevalent, but the loss of its symbionts possibly yields the loss of most of its UVR protection, potentially leading to higher prevalence of long-term cellular damage. The response to UVR, combined with the interaction of temperature and bleaching, and its effect on sperm development, motility, mitochondria, and DNA damage should be investigated.

In order for sperm limitation to not hinder successful reproduction of benthic broadcast spawning marine invertebrates, fertilization is dependent upon, among other factors, population size, density, and spawning synchronization^79,94,95^. As one of the two most common reef-building corals in Kāneʻohe Bay, *M. capitata* is found in high density and across a variety of depths and reef habitats^51,52^. Furthermore, since *M. capitata* is highly synchronized in its gametogenesis and spawning behavior^59,60^ and has more gametes per bundle than *Montipora spp*. (Table 1, Fig. 2), then its successful reproduction might not be impeded by a higher percentage of low motile sperm in high density populations.

Prior to anthropogenic disturbance increasing the frequency and severity of thermal stress events, these adaptations and life-history traits were likely well-suited for each species in its habitat. However if, as predicted, large-scale bleaching events continue to occur more frequently^19,24^, *Montipora spp*. colonies that experience high mortality might not have enough time to regrow to sufficient colony size to sexually reproduce, even though they may have the potential to recover more quickly. Likewise, if *M. capitata* takes four or more years to fully recover, frequently recurring warming events could feasibly negatively impact its capacity for successful reproduction if mortality pushes some populations to a point that they become limited by colony density whereupon a decline in sperm motile energy exacerbates the negative effects of gamete concentration on successful fertilization. As previously stated, most pre-zygotic studies of bleaching and coral reproduction detail oocyte development, size, and energy investment, with minimal attention to sperm, save histological measurements and counts. Yet arguably the most critical function of sperm physiology and evolution—its ability to swim—is rarely considered. Investigating and tracking both aspects of reproductive physiology and their response to environmental stress will allow for a more complete understanding of the capacity—or inability—of coral populations to adapt to life in the Anthropocene.

## Methods

### Animal care and gamete collection

In late May and early June 2018 and 2019, a 20–30 cm fragment from colonies of both species was collected from sites across the bay (21°25′57.21″ N, 157°47′16.96″ W). Because *Montipora spp*. inhabits shallow areas of the reef, colonies for both species were selected from a maximum depth of 3–4 m. Donor colonies were originally selected that were most likely to be of reproductive age, in a size range of approximately 1–3 m^2^. One *M. capitata* and one *Montipora spp*. donor colony were each closer to ½ m^2^, and the largest *Montipora spp*. colony was ~ 10–12 m^2^. However, because large, reproductive-sized *Montipora spp*. colonies are not common in Kāneʻohe Bay (the species is not found on every patch reef) and the species is not a frequent target of investigation, the study was limited to available colonies. Since finding *Montipora spp*. was the limiting factor, each time a colony was located, it was tagged along with a nearby *M. capitata* colony. Most of the *Montipora spp*. colonies are separated by dozens (and some hundreds) of meters from their nearest conspecific and were often on separate patch reefs in the bay.

Each colony fragment was tagged with an individual number, and kept in an outdoor, flow-through seawater tank under 70% shade cloth at the Hawaiʻi Institute of Marine Biology (HIMB) on Coconut Island in Kāneʻohe Bay (2018: *Montipora spp.* N = 16, *M. capitata* N = 10; 2019: *Montipora spp.* N = 14, *M. capitata* N = 16). Flow rates were approximately 50 L/min and an average midday PAR ~ 900 μmol m^−2^ s^−1^. Small powerheads in the tanks increased water circulation, and a ‘surge tank’ was added to the *Montipora spp.* holding tanks to further mimic their high wave energy environment. The colony fragments were housed in these tanks for the summer spawning season and were inspected daily for signs of stress, tissue loss, or lesions. In the rare event that a lesion did appear, an area approximately 2–3 cm deep into apparently healthy tissue and skeleton was removed, and the area was covered with two-part epoxy.

Two hours before their predicted spawning time at 21:00, each colony was isolated to allow independent collection of egg-sperm bundles. Because *Montipora spp.* spawning dates were uncertain, colonies were monitored nightly from late May through early September in 2018 and nightly in June and July in 2019. If a colony had not spawned by 22:00, it was placed back in the holding tank.

Even though animals were not subject to IACUC certification, colonies were checked every day, and all efforts were made to ensure high quality conditions in the tanks to reduce stress and support reproductive health. After the spawning season, coral fragments were returned to the original locations and secured back onto the reef using two-part epoxy. Collection of corals was approved under Special Activity Permit Numbers SAP 2018–03, SAP 2019–16, and SAP 2020–25 and issued by the State of Hawaiʻi Department of Land and Natural Resources.

### Eggs and sperm concentration per bundle

In 2018, the number of eggs and sperm concentration per bundle were assessed for colonies of both *M. capitata* (N = 8) and *Montipora spp.* (N = 12). A single egg-sperm bundle was collected in 50 μL of seawater with a 200 μL pipette and placed in 400 μL of 0.2 µm filtered seawater in an Eppendorf tube, repeated to collect 10 total bundles per colony; later 50 μL of 32% paraformaldehyde was added to preserve the sperm in a total volume of 500 μL, for a final concentration of 3.2% paraformaldehyde in filtered seawater. Eggs per bundle (bundles: n = 156 M*. capitata*, n = 156 *Montipora spp.*) were counted the night of the spawn using a Wild M3 dissecting microscope (Leica Microsystems, Buffalo Grove, IL) at 25 × magnification. The preserved sperm (bundles: n = 142 M*. capitata*, n = 142 *Montipora spp*.) were later counted using a hemocytometer (Thermo Fisher Scientific, Waltham, MA) with an Olympus BX41 microscope (Center Valley, PA). One colony of *M. capitata* and some of the egg-sperm bundles from other colonies of both species were aspermic, leading to fewer bundles assessed for sperm than eggs.

### Egg volume

In 2019, mean egg volume for *M. capitata* (n = 564; N = 16) and *Montipora spp.* (n = 564; N = 14) was measured by capturing images of approximately 30 eggs per colony on the night of spawning (microscope: Olympus SZX12, Center Valley, PA; camera: S01-0801A, software: SSView v. × 64, 1.0.5969, Motic Instruments USA, Schertz, TX). After the bundles had broken apart, the eggs were transferred to a new container with 0.2 µm filtered seawater and allowed to hydrate and expand for at least 30 min. To obtain the highest resolution possible, fewer than 10 eggs were captured in each image. The volume of the eggs was later measured using ImageJ processing software (v. 1.51a, Bethesda, MD) and a micrometer to set the appropriate scale. Eggs that were irregularly shaped or malformed were rarely found but also not used in the analysis.

### Sperm motility with computer assisted sperm analysis (CASA)

Sperm motility was assessed with Computer Assisted Sperm Analysis (CASA) software using computer-aided video microscopy (CASA: Hamilton Thorne, Beverley, MA; microscope: Olympus BX41, Center Valley, PA). To prevent the need for multiple serial dilutions, egg-sperm bundle collection targeted the ideal sperm concentration (ca. 5 × 10^6^ to 5 × 10^7^ cells/mL) for the specialized fixed depth, 20 µm counting chambers (Leja Products BV, The Netherlands). Transfer pipettes were used to carefully collect the bundles in minimal seawater, and samples were then diluted with filtered seawater to the desired volume. Hawaiian *Montipora* corals have a toxin (found both in the adult tissue and eggs) that can immediately kill sperm if an egg is ruptured in a small volume of water^96^. Due to concerns for the toxin and desired starting concentrations for CASA, egg-sperm bundles were collected in 15 mL tubes with 100 bundles in 10 mL of seawater for *M. capitata* (2018: n = 55, N = 9; 2019: n = 23, N = 16) and 100 bundles per 5 mL of seawater for *Montipora spp*. (2018: n = 137, N = 16; 2019: n = 61, N = 14). If fewer bundles were released (primarily for *Montipora spp.*), starting seawater volume was adjusted as needed to maintain the target starting sperm concentration.

The bundles were allowed to break apart with minimal agitation, and sperm was transferred to a separate 1.5 mL Eppendorf away from the eggs. The CASA slides were loaded with 4 μL of each sperm sample, and per standard CASA software recommendation, at least five videos and a minimum of 200 sperm were recorded per sample to record total motility. If needed and depending on concentration, sperm was diluted in filtered seawater to a target of 1 × 10^7^ cells/mL. Both initial and one-hour motility measurements were taken for most samples; a new slide of the existing sample was loaded for the one-hour reassessment. CASA parameters for all samples were modified from Zuchowicz et al.^97^.

### Mitochondrial membrane potential with flow cytometry

The percentage of sperm with high mitochondrial membrane potential was analyzed using the JC-1 stain (T3168, Thermo Fisher Scientific, Waltham, MA) in conjunction with flow cytometry for *M. capitata* (2018: n = 55, N = 9; 2019: n = 23, N = 16) and *Montipora spp*. (2018: n = 50, N = 16; 2019: n = 61, N = 14). Alongside CASA motility analysis, samples were diluted with filtered seawater, if necessary, to a sperm concentration of approximately 1–5 × 10^6^ cells/mL for flow cytometry analysis, typically using 50 μL of the sperm sample and 450 μL of filtered seawater; 2.5 μl of JC-1 solution was added to 500 μL of sperm sample, incubated in the dark for 15 min, and then analyzed on a flow cytometer (BD Accuri C6 Plus, BD Biosciences, San Jose, CA). A negative control was made for each species for each night of spawning using 500 μL of sperm sample with 1 μL CCCP (carbonyl cyanide 3-chlorophenylhydrazone, C2759, Millepore-Sigma, St. Louis, MO). After at least 15 min with CCCP, the negative control was then incubated with 2.5 μL JC-1 in the dark for another 15 min and then also analyzed on the flow cytometer. The negative control was later used to assist with sample gating analysis to help identify the population of unhealthy or dead sperm cells. Unstained control samples (500 μL) for each individual coral that spawned each night were also run through the flow cytometer to ensure that there was no other auto-fluorescence interference. The flow cytometer has an argon laser with excitation at 488 nm, and channels measured fluorescence at plots on two wavelengths: green FL1 (533/30 nm) and orange FL2 (585/40 nm). The JC-1 dye will emit green fluorescence at low membrane potential and a higher proportion of orange fluorescence at high membrane potential. BD Accuri C6 Plus software (version 1.0.23.1, BD Biosciences, San Jose, CA) was used for sample gating and analysis.

### Fertilization

Fertilization trials for *M. capitata* (2018: n = 14, N = 12; 2019: n = 19, N = 19) and *Montipora spp*. (2018: n = 18, N = 13; 2019: n = 30, N = 26) were conducted via bundle-bundle crosses: adding two egg-sperm bundles (one bundle each from two individual colonies) into 5 mL of 0.2 µm filtered seawater in 20 mL scintillation vials, yielding an approximate sperm concentration of 3–6 × 10^5^ cells/mL. After the bundles broke apart, the contents were gently mixed and allowed to sit for 60 min at 26 °C, during which the total number of eggs per vial was counted. One hour after the bundles had broken apart, the eggs were rinsed twice in 10 mL filtered sea water to remove sperm and then filled with 15 mL filtered sea water. After three hours, the number of developing, fertilized eggs per vial were counted under a Wild M3 dissecting microscope at 10 × magnification. Selfing controls were performed by adding two bundles from the same colony to 5 mL of filtered seawater in a scintillation vial and analyzing the number of fertilized eggs after three hours.

### Settlement

Developing larvae were kept in glass bowls with ~ 100 mL filtered seawater at 26 °C and allowed to develop for 7–10 days; water was changed daily with 0.2 µm filtered seawater during development. For settlement, larvae were pooled and placed in six-well plates at a density of 10 larvae per well: *M. capitata* (2018: n = 36, N = 7; 2019: n = 18, N = 19) and *Montipora spp*. (2018: n = 56, N = 19; 2019: n = 55, N = 24). Each well was filled with 10 mL of filtered seawater, and a small, 1 cm chip of crustose coralline algae (CCA) was added to provide settlement cues. The CCA species used for settlement, *H. reinboldii*, can mediate larval settlement^98,99^ and was collected near some of the donor colonies at less than 10 m depth. Because *M. capitata* can have a lengthy pelagic larval duration^98^, larvae were allowed several weeks to settle, and seawater was changed in each well every other day. Each well was periodically assessed using a dissecting microscope at 10 × magnification and spat recorded if settled. After one month, final settlement counts were taken.

### Statistical methods

Where necessary, data were transformed using the appropriate transformation method in order to meet test assumptions, and normality assumptions were determined visually or with a Shapiro–Wilk’s test. Additionally, the possibility that unequal sample size influenced the results in some experiments was eliminated by subsampling to similar sample size at random 10 times to confirm that results did not change. Data for sperm per bundle were transformed with a Box–Cox transformation. Eggs per bundle data were analyzed using a *t* test with Welch’s correction, and sperm concentration per bundle was analyzed with a *t* test. Egg volume data were square-root transformed and analyzed with a *t* test with Welch’s correction.

The data for both sperm percent total motility and percent of sperm with high membrane potential were arcsine transformed for correlation analysis, and a two-way ANOVA was used to investigate the interaction between species and year with a Tukey’s post hoc to determine differences among each. The comparison of the initial and one-hour motilities for species/year were each analyzed using a *t* test with Welch’s correction. The *M. capitata* 2018 initial and one-hour motility motilities were square root transformed to better meet test assumptions.

Percent fertilization data were arcsine transformed, and percent settlement data were square root transformed. For both, a two-way ANOVA was used to investigate the interaction between species and year with a Tukey’s post hoc to determine differences among each. Map was generated using *Google Earth Pro* v. 7.3.3.7786. GraphPad Prism 9 software (version 9.0.1; San Diego, CA, USA, www.graphpad.com) was used to analyze gametes per bundle, egg volume, initial and one-hour motilities, correlation analysis of sperm motility with membrane potential, and to create all graphs. Two-way ANOVAs for sperm motility, membrane potential, fertilization, and settlement were analyzed with R version 3.5.3^100^.

## Supplementary Information

Supplementary Data

Supplementary Information
