# Supplementary material for: Reproductive plasticity of Hawaiian Montipora corals following thermal stress
Source: Sci Rep. 2021 Jun 9;11:12525. doi: 10.1038/s41598-021-91030-8 (PMC8190081; doi:10.1038/s41598-021-91030-8)
Supplement: Supplementary file 2 — Supplementary Information [file 41598_2021_91030_MOESM2_ESM.pdf]

Supplementary Information for

Reproductive plasticity of Hawaiian *Montipora* corals following thermal stress

E.M. Henley, M. Quinn, J. Bouwmeester, J. Daly, N. Zuchowicz, C. Lager, D.W. Bailey, and M. Hagedorn

Send correspondence to: henleym@si.edu or emhenley@hawaii.edu

This file includes:
Tables S2 to S5, results of two-way ANOVAs for:
- Percent sperm motility
- Percent of sperm with high mitochondrial membrane potential
- Percent fertilization
- Percent settlement

Figure S1 – results of ANOVA comparison of percent fertilization for *M. flabellata* x *M. flabellata*; *M. flabellata* x *M. dilatata*; *M. dilatata* x *M. dilatata*

Additional supplementary information for this manuscript:
"Supplementary Data Set File_All Data" is an Excel data file that includes the data for this manuscript. Each tab in the Excel file corresponds to data for:
1. Egg and sperm per bundle
2. Egg volume
3. Percent sperm motility and percent high mitochondrial membrane potential
4. Percent sperm motility initial and 1-Hr
5. Percent fertilization
6. Percent settlement
7. Preliminary percent sperm motility data from 2017
8. Percent fertilization comparisons for *M. flabellata* x *M. flabellata*; *M. flabellata* x *M. dilatata*; *M. dilatata* x *M. dilatata*

Table S2. Two-way ANOVA for percent total sperm motility.

	Sum Sq.	DF	F-value	p-value
(Intercept)	13.53	1	303.134	< 2e-16 ***
Species	8.5412	1	189.338	< 2e-16 ***
Year	0.6482	1	14.521	0.000189
Species: Year	1.6354	1	36.639	7.7e-09 ***
Residuals	8.2576	185		

There is a significant interaction between species and year ($F = 36.639$, $p < 0.0001$). Species ($F = 189.34$, $p < 0.0001$) and year ($F = 14.52$, $p = 0.0002$) are both significant factors, with species have a stronger effect.

Table S3. Two-way ANOVA for percent of sperm with high mitochondrial membrane potential.

	Sum Sq.	DF	F-value	p-value
(Intercept)	7.9678	1	280.04	< 2.2e-16 ***
Species	7.4753	1	262.73	< 2.2e-16 ***
Year	1.7487	1	61.46	3.463e-13 ***
Species: Year	0.7349	1	25.83	9.078e-07 ***
Residuals	5.2638	185		

There is a significant interaction between species and year ($F = 25.8$, $p < 0.0001$), and both species ($F = 262.73$, $p < 0.0001$) and year ($F = 61.46$, $p < 0.0001$) are significant factors.

Table S4. Two-way ANOVA for percent fertilization.

	Sum Sq.	DF	F-value	p-value
(Intercept)	4.2453	1	74.2515	6.572e-13***
Species	0.0334	1	0.5845	0.4468842
Year	0.2642	1	4.6202	0.000101 ***
Species: Year	0.7221	1	12.6304	0.0006522***
Residuals	4.4025	77		

There is a significant interaction between species and year ($F = 12.63$, $p = 0.0006$), and year is a factor ($F = 4.62$, $p = 0.0001$) with the greatest significance. Species did not have a significant effect ($F = 0.5845$, $p = 0.4469$).

Table S5. Two-way ANOVA for percent settlement.

	Sum Sq.	DF	F-value	p-value
(Intercept)	0.74094	1	58.3281	2.488e-10***
Species	0.10095	1	7.9471	0.0065786**
Year	0.20055	1	15.788	0.0001984***
Species: Year	0.21774	1	17.1409	0.0001139 ***
Residuals	0.73677	58		

There is a significant interaction between species and year ($F = 17.1409$, $p = 0.0001$), and there are significant main interactions with both species ($F = 7.947$, $p = 0.0066$) and year ($F = 15.79$, $p = 0.0002$).

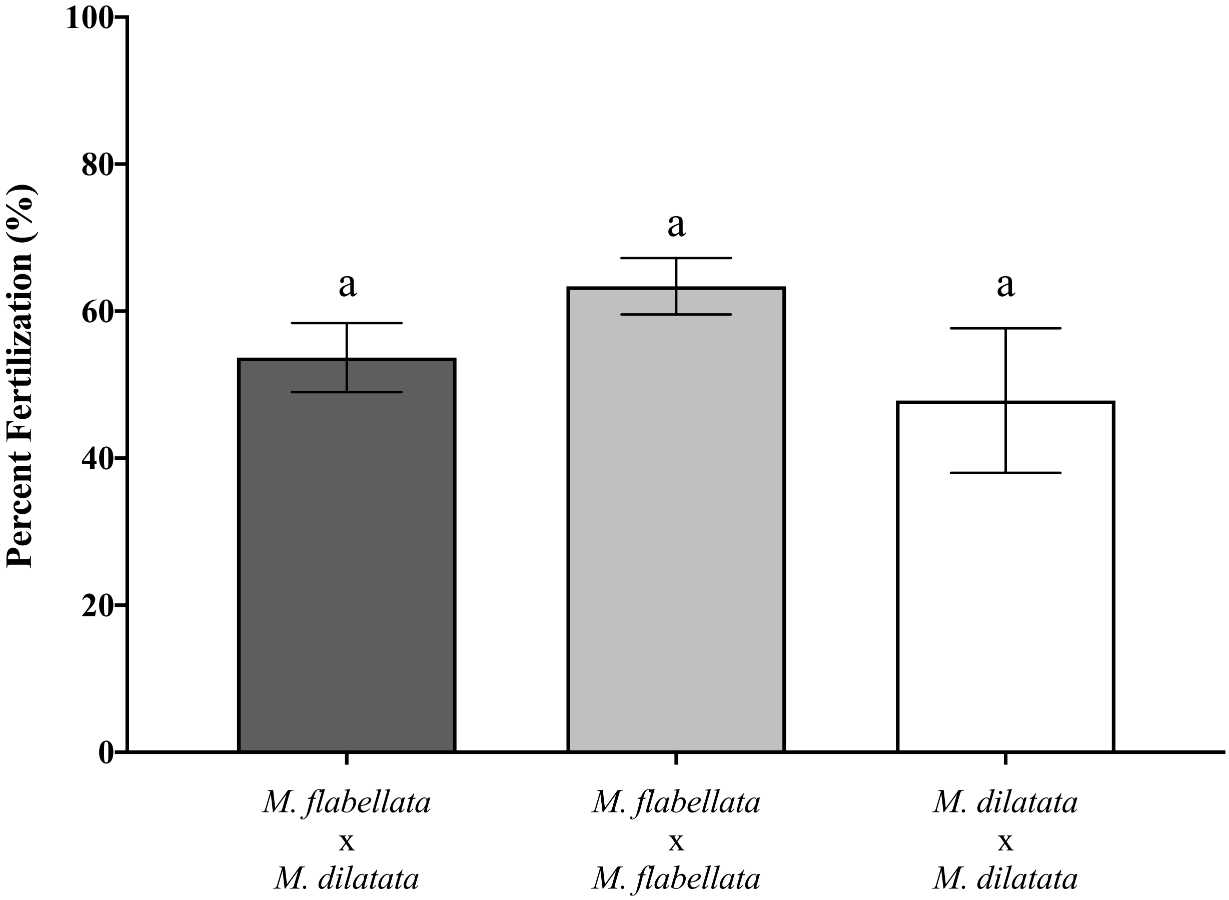

Figure S1. Percent fertilization for *M. flabellata* and *M. dilatata* crosses.
There was no difference in the mean percent fertilization of crosses with corals identified as *M. flabellata* x *M. dilatata* (N = 14 unique crosses; n = 17 fertilization trials), *M. flabellata* x *M. flabellata* (N = 19 unique crosses; n = 26 fertilization trials), and *M. dilatata* x *M. dilatata* (N = 5 unique crosses; n = 5 fertilization trials). Fertilization data for each cross is combined from both 2018 and 2019; one-way ANOVA results: $F_{(2,45)} = 2.041$, $p = 0.1418$. Because of these similarities in fertilization, all *M. flabellata* and *M. dilatata* colonies were combined to a common *Montipora spp*. for comparison with *M. capitata*.
